# Integrative analysis of tissue-specific methylation and alternative splicing identifies conserved transcription factor binding motifs

**DOI:** 10.1093/nar/gkt652

**Published:** 2013-07-24

**Authors:** Jun Wan, Verity F. Oliver, Heng Zhu, Donald J. Zack, Jiang Qian, Shannath L. Merbs

**Affiliations:** ^1^Department of Ophthalmology, Wilmer Institute, Johns Hopkins University School of Medicine, 600 North Wolfe Street, 21287 Baltimore, MD, USA, ^2^Department of Pharmacology and Molecular Science, Johns Hopkins University School of Medicine, 600 North Wolfe Street, 21287 Baltimore, MD, USA, ^3^Department of Molecular Biology and Genetics, Johns Hopkins University School of Medicine, 600 North Wolfe Street, 21287 Baltimore, MD, USA, ^4^Department of Neuroscience, Johns Hopkins University School of Medicine, 600 North Wolfe Street, 21287 Baltimore, MD, USA, ^5^Institute of Genetic Medicine, Johns Hopkins University School of Medicine, 600 North Wolfe Street, 21287 Baltimore, MD, USA and ^6^Institut de la Vision, 17 rue Moreau, 75012 Paris, France

## Abstract

The exact role of intragenic DNA methylation in regulating tissue-specific gene regulation is unclear. Recently, the DNA-binding protein CTCF has been shown to participate in the regulation of alternative splicing in a DNA methylation-dependent manner. To globally evaluate the relationship between DNA methylation and tissue-specific alternative splicing, we performed genome-wide DNA methylation profiling of mouse retina and brain. In protein-coding genes, tissue-specific differentially methylated regions (T-DMRs) were preferentially located in exons and introns. Gene ontology and evolutionary conservation analysis suggest that these T-DMRs are likely to be biologically relevant. More than 14% of alternatively spliced genes were associated with a T-DMR. T-DMR-associated genes were enriched for developmental genes, suggesting that a specific set of alternatively spliced genes may be regulated through DNA methylation. Novel DNA sequences motifs overrepresented in T-DMRs were identified as being associated with positive and/or negative regulation of alternative splicing in a position-dependent context. The majority of these evolutionarily conserved motifs contain a CpG dinucleotide. Some transcription factors, which recognize these motifs, are known to be involved in splicing. Our results suggest that DNA methylation-dependent alternative splicing is widespread and lay the foundation for further mechanistic studies of the role of DNA methylation in tissue-specific splicing regulation.

## INTRODUCTION

DNA methylation plays an important role in the epigenetic regulation of gene expression; yet, the exact mechanisms by which DNA methylation affects transcriptional regulation are not fully understood. Approximately 20% of CpGs are located in clusters (CpG islands) within the genome (1). A similar number of CpG islands are found in both the human and mouse genomes (2). The majority of CpG islands are in promoters of annotated genes and are generally unmethylated, a state that correlates with expression of the downstream gene. The remaining CpG islands are dispersed between intra- and intergenic regions (2); however, unlike the CpG islands that overlap promoters, methylation of these regions is not necessarily associated with gene silencing (3).

Previous studies have shown that tissue-specific methylation occurs largely at the intra- and intergenic CpG islands (3–6). During development, *de novo* methylation at intra- and intergenic CpG islands has been hypothesized to play an important role in tissue differentiation by controlling gene expression in a time-dependent manner (2). This hypothesis is supported by evidence suggesting that >40% of intra- and intergenic CpG islands in the human and mouse overlap with sites of transcription initiation (2). CpG-containing regions that do not qualify as CpG islands [defined as regions ≥200 bp with a GC content >50% and an observed:expected CpG ratio of >0.6 (1)] also appear to be important in regulating gene expression. For example, areas located adjacent to CpG islands, [within 3 kb, ‘CpG shores’, or 4 kb ‘CpG shelves’ (7)], have been used to identify regions of both tissue-specific and disease-associated differential methylation (3,7–15).

Alternative splicing is an important mechanism for maximising variation in expression from a single genome, especially in a tissue-specific manner. We and others have found that intragenic DNA methylation appears to regulate tissue-specific expression from alternative promoters (6,16,17). Distinctive intragenic epigenetic patterns, including nucleosome positioning, DNA methylation and histone modifications, have been identified around exons and exon–intron borders, suggesting that chromatin structure is also important to exon selection (18–25).

The first experimental example of intragenic DNA methylation regulating alternative splicing was described for the *CD45* gene (26). At this locus, CTCF transcription factor (TF) binding is associated with RNA Polymerase II pausing and exon 5 inclusion. Mechanistically, CTCF binding to DNA appears to create a roadblock to Pol II elongation, slowing the transcription process and enabling the inclusion of a weak exon. CTCF binding is blocked by DNA methylation (27), and when DNA methylation blocks CTCF binding downstream of the alternatively spliced (AS) exon, the weak exon is skipped (26). The CTCF findings suggest that there may be additional, as yet unidentified, DNA methylation-dependent splicing mechanisms that are contingent on spatial cues.

These studies emphasize the need for an unbiased analysis of genome-wide DNA methylation to identify patterns regulating tissue-specific alternative splicing. We previously performed a detailed survey of DNA methylation on the majority of CpG sites in the mouse genome, comparing the genome-wide DNA methylation patterns of two related, but distinct neurological tissues: retina and brain. Tissue-specific differentially methylated regions (T-DMRs) were identified with the MeKL-chip method, using MBD2b/MBD3L1-enrichment with kinase-modified ligation mediated PCR-and subsequent hybridization to the comprehensive high-throughput array for relative methylation (CHARM) NimbleGen tiling array (17,28). In our present study, we characterized the T-DMRs by genomic location and CpG distribution and found an overrepresentation of T-DMRs within intragenic genomic locations. These T-DMRs were highly conserved between species, suggesting an important role in genome regulation. Combined analysis of mouse retina and brain T-DMRs and our previously published transcriptome data (29) revealed that a significant number of T-DMRs are associated with AS genes, including both inclusion or exclusion of an exon, providing additional evidence for a role of DNA methylation in alternative splicing. Subsequent bioinformatic analysis reveals, for the first time, unique groups of binding motifs associated with either exon inclusion, exclusion or both. The discovery of distinct DNA-binding motifs and the requirement for specific location significantly expands our understanding of the mechanisms by which DNA methylation may influence alternative splicing.

## MATERIALS AND METHODS

### Genome-wide methylation analysis

Methylation enrichment was performed on adult (P50P56) retina and brain tissue from C57BL/6J mice (*n*=3) as previously described (17). Briefly, MBD2b/MBD3L1-enrichment (MethylCollector Ultra Kit, Active Motif) and subsequent kinase ligation-mediated PCR (MeKL) was used. Methylation-enriched DNA, alongside input DNA, was hybridized to a custom 2.1 M NimbleGen array [the CHARM platform (28)]. The R/Bioconductor software for CHARM (28,30) was used to calculate relative methylation level as compared with the input (unenriched) channel for all probes on CHARM microarray, from which T-DMRs between brain and retina were identified. Two sets of raw data were derived for each MeKL-chip microarray (untreated input DNA and methylation-enriched DNA). Lowess normalization within each sample for all control probes (30) and quantile normalization between samples was performed. The methylation level for each probe was defined as ratio of methylated probe signal to the input probe signal. The CHARM array contains some probes that do not match to any genomic sequence. Signals from these probes are considered background noise. In our analysis, the probes with input intensities lower than background were removed. Differentially methylated probes were identified using a *t*-test. We selected a cut-off of *P* < 0.005, which corresponds to false-discovery rate (FDR) of 4.6% based on a permutation analysis (28). T-DMRs must contain at least three neighboring differentially methylated probes.

### T-DMR enrichment analysis

The expected numbers of T-DMRs in different genomic regions (gene promoters, 5′UTR, exons, introns, 3′UTR and intergenic regions) are proportional to the number of probes designed within these regions. The statistical significance of T-DMRs enriched in each region was calculated using a hyper-geometric test, comparing the observed and expected numbers of T-DMRs in each genomic region.

### Gene ontology analysis

For a given group of genes of interest, we calculated the enrichment for each associated Gene Ontology (GO) term and its statistical significance (*P*-value). The *P*-value was calculated based on the hyper-geometric distribution then modified by multiple-test Bonferroni correction. Enriched GO terms were identified only if its modified *P*-value was <0.05.

### Evolutionary conservation determination

The evolutionary conservation score (PhastCons17way) was obtained from comparison with 16 other vertebrate genomes and downloaded from UCSC Genome Browser (31). We calculated the percentage of nucleotides with conservation scores greater than a given threshold. This analysis was applied to all probes on the CHARM microarray, T-DMRs and sequence motifs identified within T-DMRs.

The nucleotide sequence of T-DMR on the predicted gene 5607 (*Gm5607*) was acquired through the UCSC Genome Browser database. Orthologous regions of *Gm5607* from the human, rat, dog, chicken, opossum, chimp and rhesus genome were downloaded from UCSC Genome Browser. Sequence alignment was performed using ClustalW2 (32,33).

### Pyrosequencing validation

Pyrosequencing assays for the mouse and rat genome were designed to interrogate methylation at the three fully conserved CpG sites using the PyroMark Assay Design Software (Qiagen; Supplementary Table S1). DNA was extracted from retina and brain of four adult C57BL/6J mice, 1 µg of which was bisulfite converted as described previously (17). In addition, DNA was extracted from retina and brain (cerebellum) of three 7-week-old Lewis rats and 500 ng was bisulfite converted using the EZ DNA Methylation-Gold™ Kit (Zymo Research). PCR was performed using 1 µl of bisulfite-converted DNA and HotStarTaq DNA Polymerase (Qiagen) under the following cycling conditions: 95°C for 15 min; 45 cycles of [94°C for 30 s, annealing temperature (56.3°C for rat, 58.3°C for mouse) 30 s, 72°C for 60 s]; 72°C for 3 min; 4°C hold followed by storage at −20°C. Amplicons were analyzed on a PyroMark Q24 Pyrosequencer as per the manufacturers’ protocols, and methylation at the CpG sites was quantified using the PyroMark Q24 software version 2.0.6 (34).

### Identification of differentially spliced genes

The inclusion level of a given exon was represented by normalized intensity (NI), the expression level of the exon relative to overall expression level of the gene hosting the given exon (35). The difference between NIs [the so-called splicing index (SI)], was adopted to reflect the fold change of exon inclusion level. If an exon underwent significant change [*P* < 0.01, corresponding to FDR < 12.3%, and |SI| > log_2_(1.25)], the exon was defined as AS between retina and brain. By comparing the inclusion levels of 114 865 exons on 10 910 expressed genes between retina and brain, 8672 differentially spliced exons were identified.

### Identification of genes with alternative start sites

Genes with multiple start sites were selected from the RefGene annotation profile downloaded from UCSC Genome Browser (31). The alternative start region was defined as the location between different start sites in the same gene. A gene was denoted as containing an alternative start site only if alternative splicing between retina and brain was identified within its alternative start region via the mouse exon array.

### Identification of 6mer motifs

T-DMRs were selected and grouped based on their regulation type (negative or positive or dual) and relative location to the spliced exons (1 kb at upstream or downstream). Ten thousand exons were randomly selected, and the 1 kb upstream or downstream sequences were used as background for comparison. We enumerated all possible 6mers to compare their occurrence in T-DMR groups to corresponding background sequences. The significance of each 6mer was evaluated using binominal model such that:

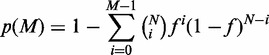

Where *M* is occurrence number of the motif, *N* is total number of all 6mers in the foreground (T-DMR groups), and *f* is the frequency of the motif in the background (all 10 000 random exon upstream/downstream sequences). All *P*-values were modified by Benjamini and Hochberg FDR multiple-test correction. One percent of FDR was selected as the threshold for significant motifs.

### Prediction of putative TFs that bind to the predicted motifs

Similar 6mers were clustered into consensus sequences. We compared the predicted consensus sequences with consensus sequences from both UniPROBE (36) and TRANSFAC (37) using a motif comparison tool (Tomtom) (38).

### Calculating motif pair distance

Pair-wise distances of motifs identified in the T-DMR(s) located within 1 kb either upstream or downstream of the same AS exon was calculated. As a comparison, the positions of motifs in the sequences were shuffled 100 times to obtain the random distances of paired motifs.

## RESULTS

### T-DMRs are overrepresented in exons and introns of protein-coding genes

**Figure 1.**
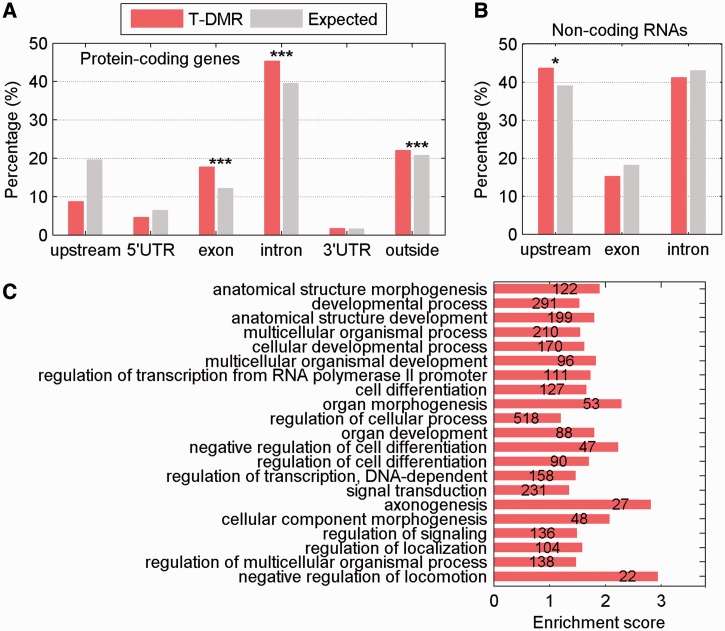
Genomic distribution and GO analysis of T-DMRs. (**A**) Percentage of T-DMR probes (red) and all probes (gray) at each location with respect to protein-coding gene position. Upstream includes up to 4 kb from the TSS. (**B**) Percentage of T-DMRs and all probes located in upstream, exon or intron regions of non-coding RNA genes. (**C**) GO analysis of all T-DMRs, the number of genes in each category is noted. Asterisks note significant enrichment (*P < 0.01; ***P < 0.0001).

DNA methylation profiling of two adult mouse tissues, retina and brain, was conducted using MBD2b/MBD3L1-enrichment and a custom CHARM tiling array (MeKL-chip) and yielded 2498 T-DMRs (17,28). These T-DMRs encompassed 17 965 50-bp probes, just 0.8% of all probes on the CHARM array. To assess the relative location profile of the T-DMRs, they were defined as either intragenic (≤4 kb upstream of the transcription start site (TSS), 5′UTR, coding exon, intron and 3′UTR) or intergenic (all other genomic locations). In total, 22.0% of the 2498 T-DMRs identified between mouse retina and brain were intergenic (i.e. outside of protein-coding genes), demonstrating a significant overrepresentation compared with percentage of all probes within the intergenic region based on the CHARM array design (20.7%; *P* = 6.4 × 10^−^^6^). The majority of T-DMRs were associated with intragenic regions (i.e. protein-coding genes; 78.0%, n = 1596). As might be expected from their proposed regulatory roles, 8.7 and 4.6% of T-DMR probes were in the upstream and 5′UTRs of genes, respectively (Figure 1A). However, this represented fewer probes than expected when compared with the CHARM array probe distributions of upstream and 5′UTR locations (19.6 and 6.4%, respectively). Similarly, T-DMRs were not overrepresented at 3′UTRs. Strikingly, the majority of gene-associated T-DMR probes were located in exons (17.8%) and introns (45.3%), which were significantly overrepresented (12.2 and 39.5%, respectively; *P* = 0, Figure 1A), suggesting that DNA methylation might play an important role in regulating alternative splicing.

We then focused on the non-coding genes that are potentially regulated by T-DMRs. The non-coding RNAs had accession names that started with the prefix ‘NR’ in RefGene. Based on the database annotations, these non-coding transcripts include structural RNAs, transcribed pseudogenes and microRNAs. On the CHARM array, 84 555 probes (3.9%) were associated with non-coding RNAs (ncRNAs), of which 690 probes (0.8 of the 3.9%) were located within a T-DMR. In contrast to protein-coding genes, T-DMRs in ncRNA genes were overrepresented in upstream regions (≤4 kb of the TSS; *P* = 7.2 × 10^−^^3^) rather than in exon and intron regions (Figure 1B). The difference in T-DMR location patterns between protein-coding genes and ncRNAs suggests that their regulation may occur through independent mechanisms.

### GO and evolutionary conservation analyses suggest functionality of T-DMRs

GO analysis of those genes containing a T-DMR showed enrichment for various biological processes, particularly development and organogenesis (Figure 1C), which is consistent with the notion that DNA methylation plays an important role in development.

**Figure 2.**
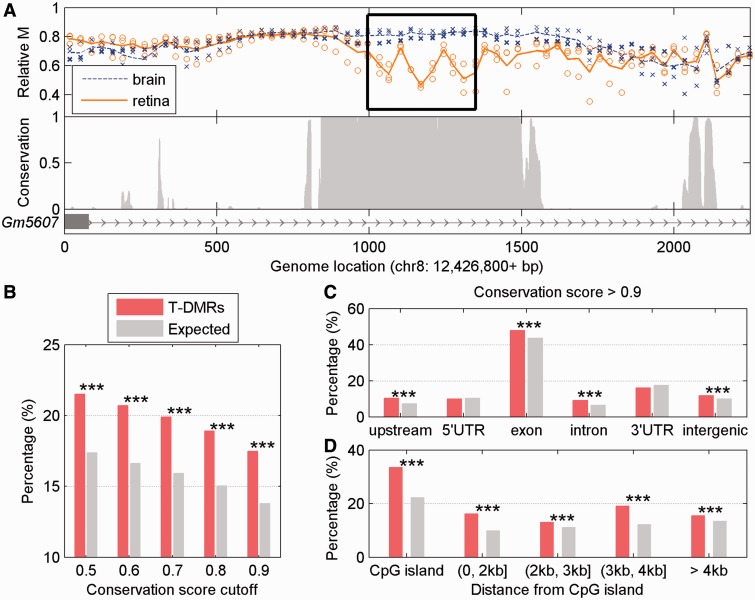
Evolutionary conservation of T-DMRs. (**A**) MeKL-chip CpG site methylation profiling (relative methylation, M) in brain (blue) and retina (orange) in the intergenic region of *Gm5607* (top) and sequence conservation across 16 species (bottom). Each point is the relative methylation at 1 probe from 1 sample; blue and orange lines show the average methylation of triplicates. The T-DMR is surrounded by a black box. (**B**) Percentage of conserved nucleotides in T-DMR (red), and all probes based on array design (gray) defined by different conservation cut-off scores. (**C**) Percentage of conserved nucleotides in T-DMRs in different gene location. (**D**) Percentage of conserved nucleotides in T-DMRs relative to nearest CpG islands. Asterisks note significance (***P < 0.0001).

The evolutionary conservation of the sequences underlying T-DMRs in 16 other vertebrate genomes was evaluated. T-DMRs tended to locate in highly conserved regions, which were surrounded by areas of lower conservation. An example of a highly conserved sequence containing a T-DMR is shown at the *Gm5607* gene (Figure 2A). Pyrosequencing of the highly conserved region, including three fully conserved CpG sites (Supplementary Figure S1), confirmed the T-DMR between retina and brain in both the mouse and the rat (Supplementary Figure S2). This high level of conservation suggests that these specific sites may be important for *Gm5607* regulation.

Globally, the percentage of nucleotides within T-DMRs with higher conservation (scores exceeding a given conservation score cut-off, e.g. 0.5) was greater than all nucleotides represented as probes on the CHARM microarray (*P* = 1.5 × 10^−^^8^; Figure 2B). T-DMRs in the upstream, exon, intron and intergenic regions were more evolutionary conserved than expected (*P* = 4.8 × 10^−9^; Figure 2C). However, no correlation was found between conservation score and absolute value of methylation difference for T-DMRs identified. As both CpG islands and island shores are important for gene regulation, we also examined the evolutionary conservation of T-DMRs overlapping these regions. The evolutionary conservation of enrichment was observed, regardless of the distance between a given T-DMR and its nearest CpG island (Figure 2D). Taken together, the GO analysis and evolutionary conservation of the identified T-DMRs suggest that these T-DMRs are likely to be significant and functional.

### T-DMRs are overrepresented in genes with alternative splicing

**Figure 3.**
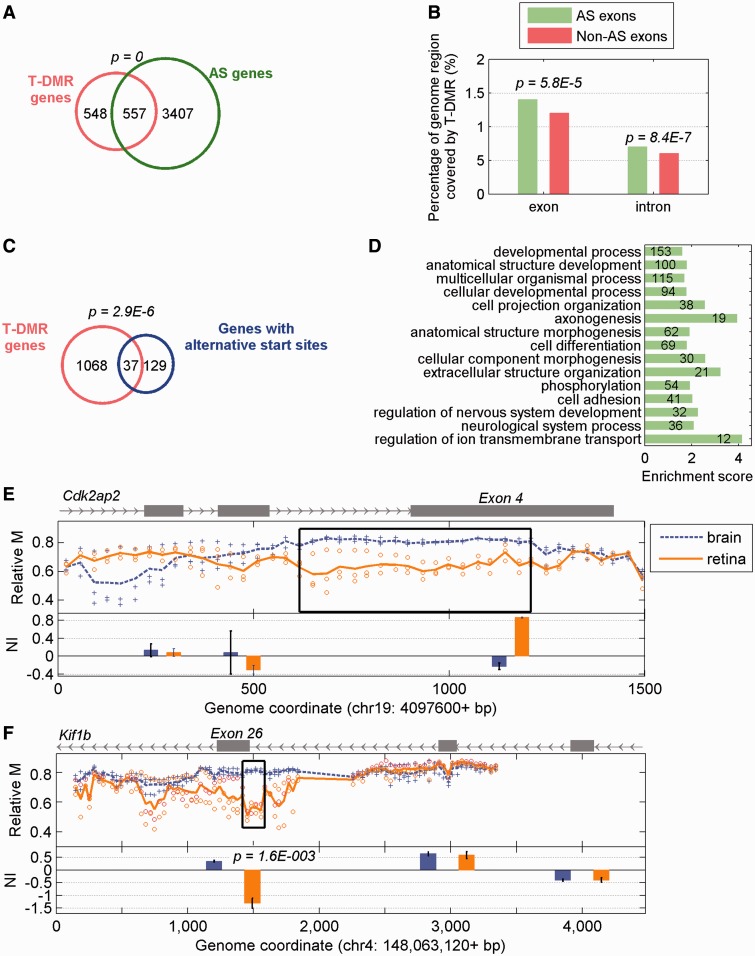
T-DMRs and genes with AS. (**A**) Venn diagram showing the overlap between all T-DMRs and those AS genes between brain and retina. (**B**) Percentage of genome location (exon and intron) covered by T-DMR for AS exons (green) and non-AS exons (red). (**C**) Venn diagram representing the overlap between all T-DMRs and those genes that showed alternative start sites between brain and retina. (**D**) Enriched GO biological processes for those spliced genes containing at least one T-DMR. (**E**) An example of methylation-dependent negative regulation on AS. Top panel shows MeKL-chip CpG site methylation profiling (relative methylation, M) of brain (blue) and retina (orange) around exon 4 in *Cdk2ap2* (see Figure 1 caption for details). A T-DMR more methylated in retina is highlighted by a black box and overlaps exon 4, which is differentially expressed (bottom panel) in the brain (blue bar) as compared with the retina (orange bar). NI is normal intensity as defined in the ‘Materials and Methods’ section. (**F**) An example of methylation-dependent positive regulation on AS, at exon 26 of *Kif1b*. A T-DMR more methylated in brain (highlighted by the black box) overlaps exon 26, which is differentially expressed in the brain as compared with the retina.

Given the large number of T-DMRs located in exons and introns and the potential functionality suggested by GO and evolutionary conservation analyses, we explored the relationship between T-DMRs and tissue-specific splicing. Using our previously published genome-wide gene expression profiling with Affymetrix exon microarrays (29), we related T-DMR location and the inclusion status of a given exon. In total, 3964 genes containing at least one differentially expressed exon between brain and retina were classified as AS. 1105 T-DMR genes were represented in the expression profiling data and over half (50.5%, n = 557) of these T-DMR-associated genes were AS (*P* = 0; Figure 3A). Conversely, ∼14% of all AS genes were associated with at least one T-DMR, whereas 8% of non-AS genes were associated with at least one T-DMR. The enrichment of AS genes containing a T-DMR was robust, regardless of the cut-off used to define the AS genes (Supplementary Figure S3). When considering only those T-DMRs located within the exon of interest and the two introns directly upstream and downstream, the AS exons tend to have higher probability of containing a T-DMRs than non-AS exons (3.7 versus 1.5%). Among the T-DMRs associated with AS exons, 138 and 191 were located in the direct upstream or downstream intron, respectively, and 95 were located in the AS exon itself. Even after normalizing by the length of the exons and introns, AS exons and their flanking introns had significantly more of their genomic sequence covered by the T-DMR than non-AS exons and introns (*P* = 5.8 × 10^−^^5^ and 8.4 × 10^−^^7^, respectively; Figure 3B). In addition, genes with alternative start sites as defined by RefSeq (see ‘Materials and Methods’ section), were significantly more likely to have a T-DMR than expected (*P* = 2.9 × 10^−^^6^; Figure 3C). Taken together, significant overlap was observed between T-DMRs and AS genes, suggesting possible role of DNA methylation in alternative splicing.

GO analysis of AS genes revealed discrete functions between AS genes with or without T-DMRs. The 557 AS genes associated with at least one T-DMR showed enrichment for genes involved in development and morphogenesis (Figure 3D). In contrast, the 3407 AS genes that were not associated with T-DMRs showed enrichment for GO terms of ‘response to stimulus’ and ‘phosphorus metabolic process’, suggesting that the DNA methylation-dependent regulation of alternative splicing occurs in a distinct set of genes.

The relationship between T-DMRs and splicing patterns between the retina and brain was further examined. Regulation was defined as ‘positive’ if relative hypermethylation in one tissue was associated with greater inclusion of an exon in the same tissue and ‘negative’ if relative hypermethylation in one tissue was associated with greater exclusion of an exon in the same tissue (i.e. greater inclusion of the exon in the other tissue). Both positive and negative regulation was observed in our analysis as shown in Supplementary Table S2. Specifically, relative hypermethylation in the brain was associated with increased inclusion in 24% of AS exons and increased exclusion of 31% of AS exons. For example, a T-DMR identified in exon 4 of cyclin-dependent kinase 2 associated protein 2 (*Cdk2ap2*) had a higher relative methylation level (increased by 0.19) in brain than in retina (Figure 3E), whereas the AS exon showed higher levels of exclusion in the brain (negative regulation). Conversely, a T-DMR overlapping the 5′ junction between an intron and the AS exon 26 was found in kinesin family member 1B (*Kif1b*), and in this case, the 0.21 greater relative methylation was associated with inclusion of the exon (positive regulation; Figure 3F). This result suggest that unlike the role of DNA methylation in gene expression, where the majority of methylation sites are repressive, intragenic DNA methylation can be equally associated with inclusion or exclusion of an AS exon.

### Distinct sequence motifs are associated with positive and negative regulation

Inspired by recent work showing that DNA methylation-dependent CTCF binding affects alternative splicing (26), we reasoned that these intragenic T-DMRs might contain binding sites for TFs and other DNA-binding proteins involved in mediating methylation-dependent alternative splicing. Therefore, we set out to identify statistically significant binding motifs that were over- or under-represented in the T-DMRs associated with AS. First, T-DMRs were divided into either positive or negative regulation groups and into either upstream or downstream groups according to their relative location to the spliced exon. Next, for each T-DMR group, a set of DNA sequences 1000 bp upstream or downstream of randomly selected exons were selected to serve as a background reference (see ‘Materials and Methods’ section). Finally, all possible 6mers in the T-DMRs were enumerated and compared with the occurrence of each motif in the T-DMRs in the corresponding background regions. The significance of each motif was then evaluated, and the significant motifs were identified (see ‘Materials and Methods’ section).

**Figure 4.**
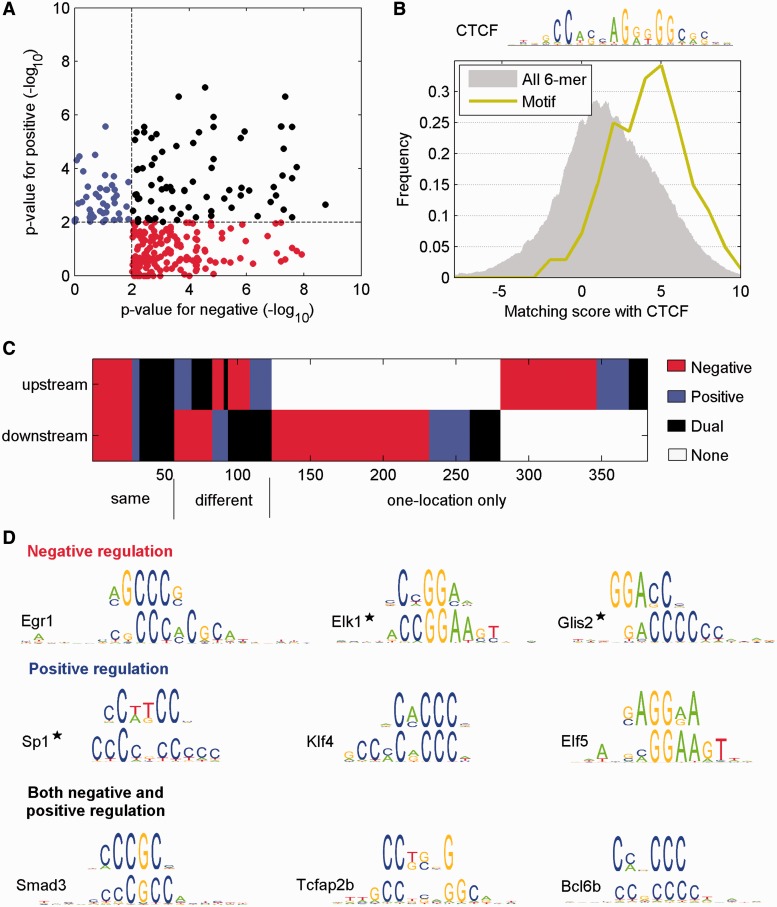
Discovery of motifs enriched within T-DMRs around AS exons. (**A**) 6mers with different methods of regulation (red: negative; blue: positive; black: both negative and positive). Each dot represents a significant 6mer. *X*- and *Y*-axis are the values of –log(p) in negative and positive group, respectively. (**B**) Similarity between significant 6mers with AS T-DMRs and the CTCF consensus sequence (shown above the graph). The gray area is the distribution of similarity scores of all 6mers (background reference) compared with the CTCF consensus sequence. (**C**) Motif regulatory roles in upstream and downstream locations relative to the AS exon. Those 6mer motifs with the same or different methods of regulation are aligned. The remaining 6mer motifs were only identified at one location. (**D**) Representative motifs identified by the study involved in positive, negative and dual regulation. The top sequences are the predicted motifs. The bottom sequences are TF-binding consensus sequences matching the motifs. The TFs marked with stars are known to interact with splicing factors. TFs not present in the expression profiles of mouse retina and brain were not considered, even if their known consensus sequences were similar to the predicted motifs.

In total, 280 significant motifs were identified within the T-DMRs downstream of AS exons (Figure 4A). Of these, 161 and 44 motifs were associated with negative and positive regulation, respectively (Figure 4A, red and blue spots, respectively). The remaining 75 motifs were associated with both positive and negative regulation (dual regulation; Figure 4A, black spots). This finding suggests that a variety of TFs, and possibly other DNA-binding proteins, might be involved in methylation-dependent regulation of AS, presumably using non-canonical mechanisms.

As CTCF has been recognized as one TF that regulates AS in co-ordination with DNA methylation (26), we examined whether the 280 motifs downstream of AS exons were enriched for the CTCF binding consensus sequence (Figure 4B). Each significant motif was compared with the CTCF consensus sequence, and a similarity score was assigned to the motif. As a control, the similarity scores from the comparison of all 6mer motifs and the CTCF consensus sequence were computed. Interestingly, the similarity scores for the CTCF consensus sequence were significantly higher for the predicted motifs than those for all possible 6mers (Figure 4B).

Similarly, we identified 224 motifs in the T-DMRs located upstream of AS exons (Supplementary Figure S4A). Of these, 116 motifs were associated with negative regulation and 54 motifs with positive regulation. An additional 54 motifs were associated with both positive and negative regulation. Increased similarity scores for the CTCF consensus sequence was also observed at these upstream T-DMRs (Supplementary Figure S4B).

Although the majority of the motifs were associated with negative regulation, irrespective of whether they were upstream or downstream of an AS exon, most motifs showed position-dependent regulation. Of the 381 unique 6mer motifs identified either downstream and/or upstream of the AS exon, 56 motifs (14.7 versus 17.3% expected; *P* = 0.1) played the same regulatory role (either negative, or positive, or dual) in both downstream and upstream positions (Figure 4C). For example, the 6mer CAGCGC was associated with negative regulation both upstream and downstream of AS exons. However, 258 6mers (67.7 versus 45.9% expected; *P* = 0) were associated with an AS exon in just one position, either upstream or downstream. For example, 6mers CCCTCA and CCCCTT, which can be recognized by CTCF, were associated with negative regulation in the downstream position but were not associated with an AS exon in the upstream position. Intriguingly, 67 6mers (17.6 versus 9.5% expected; *P* = 3.4 × 10^−^^7^) played opposing regulatory roles dependent on the relative location to an AS exon. For example, ACCGCT was associated with positive regulation in an upstream position and negative regulation in a downstream position.

Significant 6mers were hierarchically clustered by their sequence similarity, and similar 6mers constituted a consensus sequence. Based on the comparison with known TF consensus sequences and tissue-specific gene expression, we predicted potential TFs that were likely to bind to the predicted consensus sequences (see ‘Materials and Methods’ section). In addition to CTCF, we predicted a large number of putative TFs involved in AS regulation. For example, Egr1 and Elk1 were associated with negative regulation; Sp1 and Elf5 were associated with positive regulation; Smad3 and one TF AP-2 family, Tcfap2a/b/c/e were associated with both positive and negative regulation (Figure 4D).

### Identified motifs are likely to be functional

**Figure 5.**
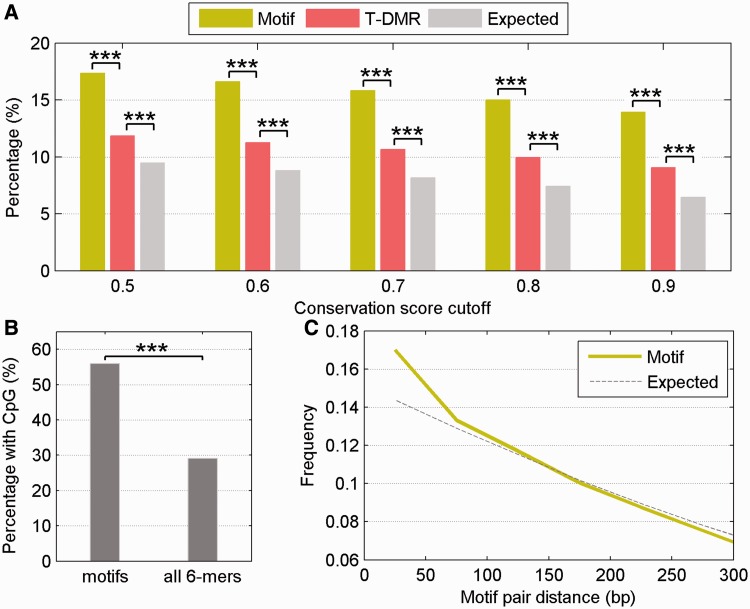
Features of the identified regulatory motifs. (**A**) The percentage of conserved nucleotides in identified motifs (green), T-DMRs (red) and genomic background (gray). (**B**) Percentage of motifs identified at AS exons near T-DMRs compared with all 6mer motifs that contained at least one CpG site. (**C**) The distribution of the pair-wise distances between motifs within T-DMRs (green line) compared with expectation (dashed gray line). Asterisks note significance (***P < 0.0001).

Several lines of evidence suggest that the predicted motifs and their corresponding TFs are likely to be functional. Although the T-DMRs already showed higher conservation than other genomic regions (Figure 2B), the predicted motifs were even more highly conserved (Figure 5A). In all, 14.0% of nucleotides within motifs had a conservation score greater than 0.9, whereas only 9.1% of nucleotides in T-DMRs reached this same conservation threshold (*P* = 3.2 × 10^−^^10^). This observation was consistent across a wide-range of conservation scores (Figure 5A).

The majority of the unique motifs (55.9%) contained at least one CpG site. In contrast, only 29.0% of all possible 6mers contained a CpG (*P* = 0, Figure 5B). Similarly, if we include all the instances of motifs in T-DMRs, the percentage of motif instances that contain a CpG is 27.0%, whereas this percentage is 12.9% in the T-DMRs (*P* = 4.2 × 10^−^^10^). The high percentage of predicted motifs containing a CpG suggests that regulation of AS through these motifs is likely to be methylation dependent.

Examining pair-wise distance between the T-DMR motifs, we found that the distribution of the distances differed from random expectation (Figure 5C). Specifically, the distances between the motifs tend to be smaller than expected, suggesting that these motifs are clustered within the T-DMRs and exert a combinatorial effect on the regulation of splicing.

Some of the TFs that recognize the T-DMR motifs are known to either regulate splicing or interact with splicing factors (Figure 4D). For example, Wilms’ tumor 1 homolog, a TF recognizing the 6mer CCCTCC, has been reported to interact with an essential splicing factor, U2AF65, and associates with the splicing machinery (39). Likewise, Elk1, a member of the ETS family of TFs, which recognizes several significant 6mers, e.g. CCGGAA/CCGGCA/CCTGGA/CCAGGA/GCCGGA, has been reported to affect AS by regulating the transcription of the splicing factor Slu7 (40).

Taken together, our analysis suggests that the predicted motifs and their corresponding TFs are likely to regulate splicing in a combinatorial and DNA methylation-dependent fashion.

## DISCUSSION

The exact function of intragenic DNA methylation and the detailed mechanisms by which it impacts transcriptional regulation remain vague. In the mouse retina and brain, we identified an overrepresentation of T-DMRs within intragenic regions of the genome, implying an important role of intragenic methylation in tissue-specific gene regulation. This role was emphasized by the fact that many of these T-DMRs were associated with genes involved in differentiation and organogenesis. Contained within these T-DMRs, we identified for the first time a complex group of predicted TF-binding motifs, which appear to both positively and negatively regulate tissue-specific alternative splicing.

Interestingly, T-DMR sequences, especially those that overlap intragenic regions of the genome, were highly conserved. Although conservation of gene body methylation, especially around exons, has been reported previously (41–43), our observation that T-DMRs identified between tissue types are conserved across species, e.g. *Gm5607*, provides further evidence that intragenic T-DMRs serve a functional role in determining tissue-specificity (42).

Although DNA methylation has recently been linked with alternative splicing (18,26,44–47), the connection between such regulation and tissue-specific alternative splicing has not been examined in detail. Examining the relationship between T-DMRs and tissue-specific alternative splicing in the retina and brain, we found that, strikingly, over half of the genes containing a T-DMR were AS, and that more than a third of AS exons directly overlapped a T-DMR. These observations indicate a wide-spread involvement of DNA methylation in tissue-specific alternative splicing. As many of the alternative spliced genes with a T-DMR were developmental genes, it is possible that these developmental genes are regulated, at least in part, by DNA methylation. In contrast, those AS genes without a T-DMR may be regulated via canonical pathways.

Non-canonical negative regulation of alternative splicing by DNA methylation has been observed for CTCF. Methylation downstream of an AS exon in *CD45* has been shown to inhibit CTCF binding, leading to the exclusion of a weak exon (26); however, our analysis found that downstream methylation of the CTCF binding motifs is also associated with positive regulation of alternative splicing. Interestingly, in about half of our T-DMRs, greater methylation was associated with exon inclusion, whereas in the other half, the converse relationship was observed. For many of the motifs, the directionality of the regulation was position dependent, with the majority of the motifs either being negative or positive regulators and present in only the upstream or downstream position. However, a smaller subset of motifs was associated with positive regulation in one position and negative regulation in the other. This complex topology of predicted motifs indicates that DNA methylation likely influences numerous TFs and multiple mechanisms to influence pre-mRNA processing. As splicing occurs co-transcriptionally (48), we hypothesize that some of these TFs will interact directly with splicing factors. Interestingly, most of our predicted motifs contain CpGs, indicating that the methylation of these motifs is likely to be biologically relevant. Determining whether methylation of these motifs inhibits or promotes the binding of TFs will require currently unavailable data sets of differential TF binding as a function of DNA methylation. Although it is generally thought that DNA methylation inhibits TF binding, recent work has demonstrated that a large number of TFs can bind to methylated DNA, suggesting the possibility that methylation can actually enhance the TF binding in some cases (49). Our results also suggest that DNA methylation can serve as a ‘switch’ for the binding status of certain TFs. In other words, the binding of TFs to these motifs is probably methylation-dependent.

Our understanding of how DNA methylation impacts gene expression has greatly expanded from the early paradigm that methylation of CpG islands in gene promoters inhibits gene expression. It is both novel and surprising that in many cases, this differential methylation appears to regulate tissue-specific alternative splicing and transcription. How methylation can result in either inclusion or exclusion of an AS exon in a gene-specific and position-specific context remains to be defined. The multiple mechanisms by which DNA methylation may affect alternative splicing have yet to be elucidated. Our identification of multiple TF binding motifs paves the way for additional experimentally based studies of the non-canonical pathways and mechanisms by which DNA methylation may modulate and regulate alternative splicing.

## ACCESSION NUMBERS

The methylation data from this study is available at Gene Expression Omnibus (http://www.ncbi.nlm.nih.gov/geo) under accession no. GSE46683.

## SUPPLEMENTARY DATA

Supplementary Data are available at NAR Online.

## FUNDING

National Eye Institute [R21EY018703 to S.M., R21EY021897 to J.Q., R01EY009769 to D.Z.]; Wilmer Core Grant [5P30EY001765 to J.Q.]; unrestricted funding from the Research to Prevent Blindness; and the generosity of A. Nixon. Funding for open access charge: Departmental funds.

*Conflict of interest statement*. None declared.

## Supplementary Material

Supplementary Data
