# Supplementary material for: Integrative analysis of tissue-specific methylation and alternative splicing identifies conserved transcription factor binding motifs
Source: Nucleic Acids Res. 2013 Jul 24;41(18):8503–14. doi: 10.1093/nar/gkt652 (PMC3794605; doi:10.1093/nar/gkt652)
Supplement: Supplementary Data [file supp_41_18_8503__index.html]

Integrative analysis of tissue-specific methylation and alternative splicing identifies conserved transcription factor binding motifs — Integrative analysis of tissue-specific methylation and alternative splicing identifies conserved transcription factor binding motifs — Supplementary Data 

# Integrative analysis of tissue-specific methylation and alternative splicing identifies conserved transcription factor binding motifs

## 

files

**Files in this Data Supplement:**

- Supplementary Data - docx file
