# Supplementary material for: Integrative analysis of tissue-specific methylation and alternative splicing identifies conserved transcription factor binding motifs
Source: Nucleic Acids Res. 2013 Jul 24;41(18):8503–14. doi: 10.1093/nar/gkt652 (PMC3794605; doi:10.1093/nar/gkt652)
Supplement: Supplementary Data [file supp_gkt652_nar-01589-x-2013-File007.docx]

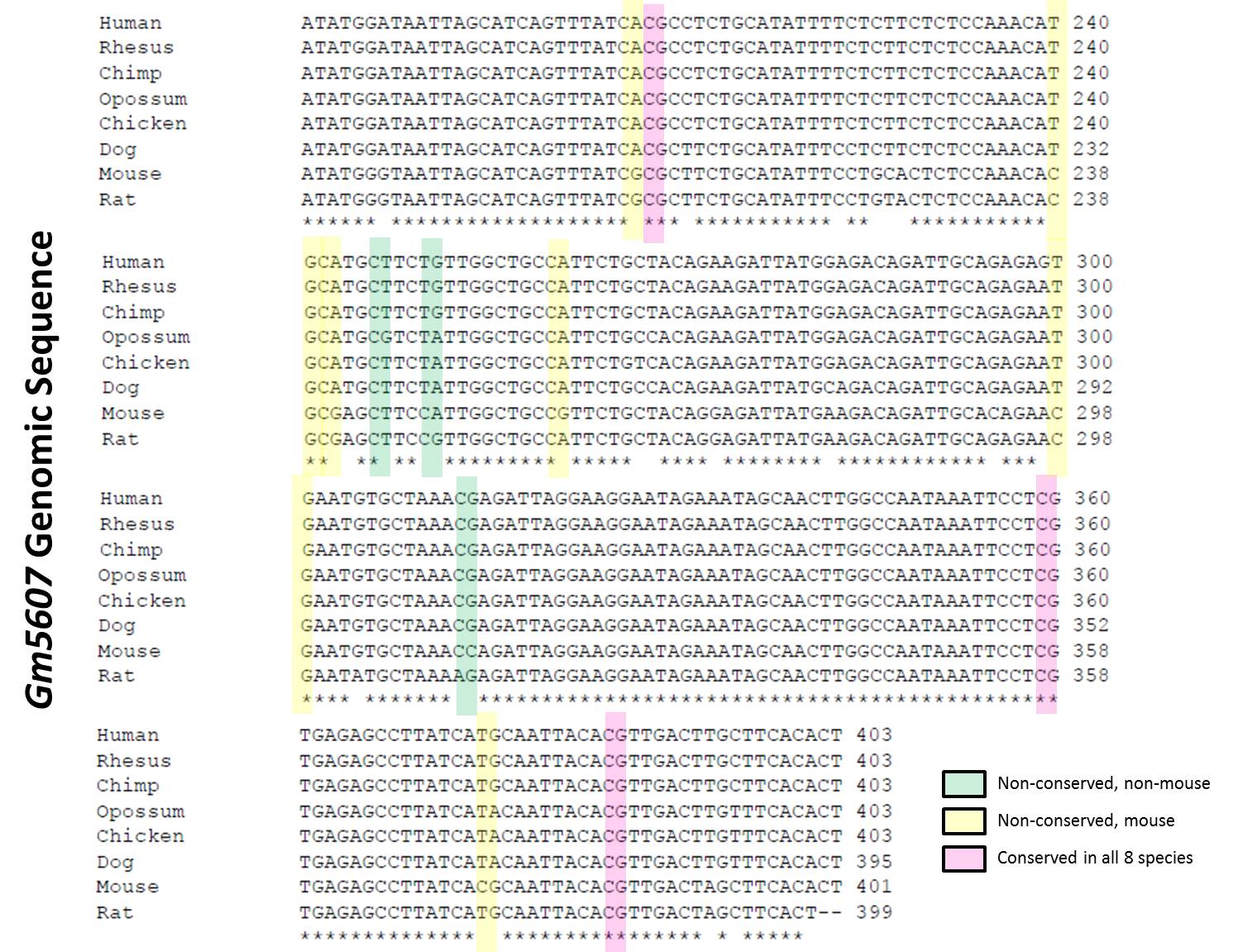


**Supplementary Figure 1.** Sequence alignment of the T-DMR within *Gm5607* (mm8, chr8: 12426800-12430300). Green columns indicated CpG sites present in non-mouse species, yellow columns indicate CpG sites present in mouse and pink columns represent CpG sites conserved in all 8 species examined.


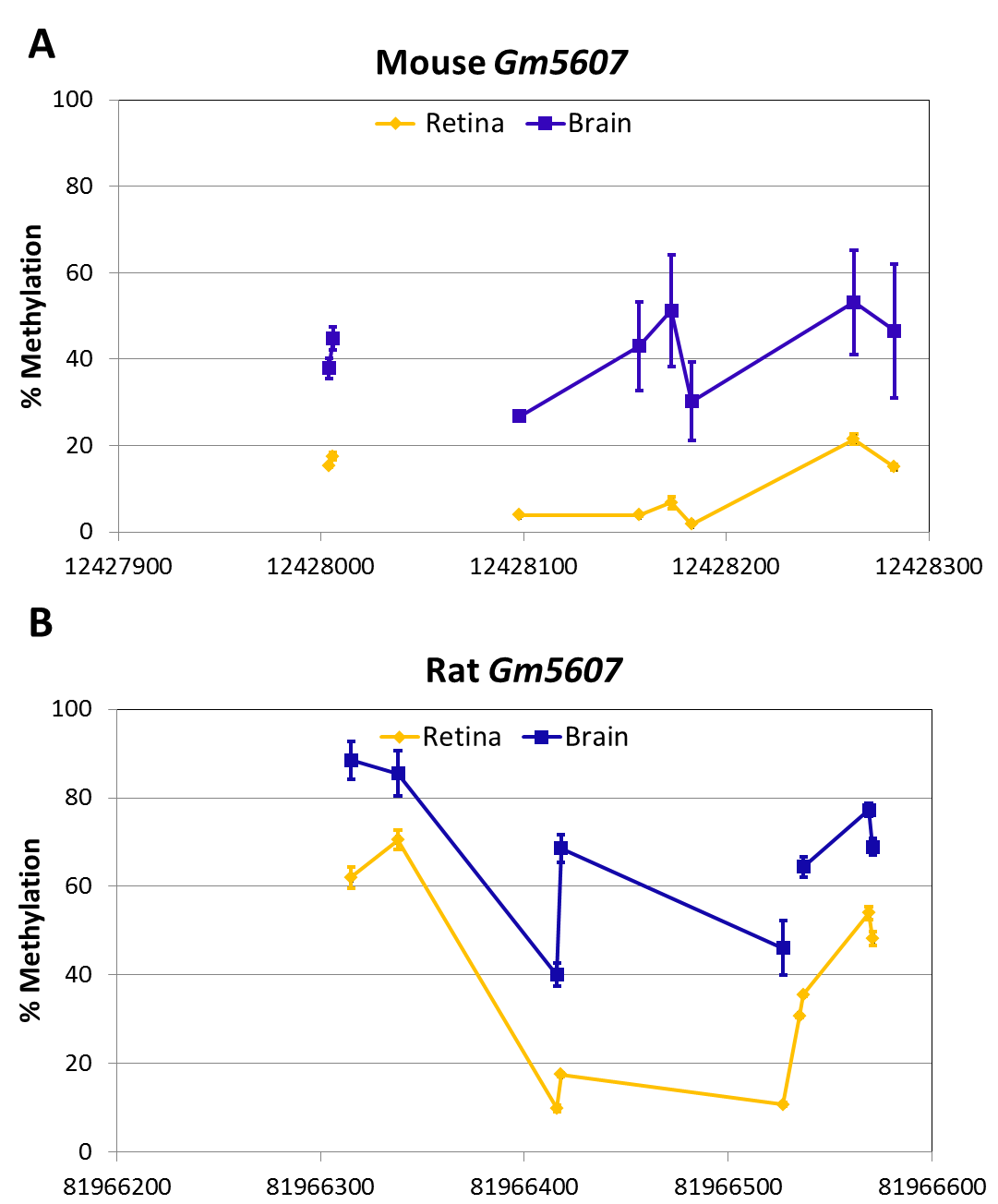


**Supplementary Figure 2.** Pyrosequencing confirming the T-DMR in mouse (A) and in rat (B). Blue represents samples from brain, yellow represents samples from retina. The lines join data from adjacent CpG sites. Error bars represent the standard deviation from biological replicates. The fully conserved CpG sites are located in mouse at (mm8) chr8: 12428006, 12428157 and 12428183 and in rat (Baylor 3.4/rn4) chr16: 81966416, 81966418 and 81966569.


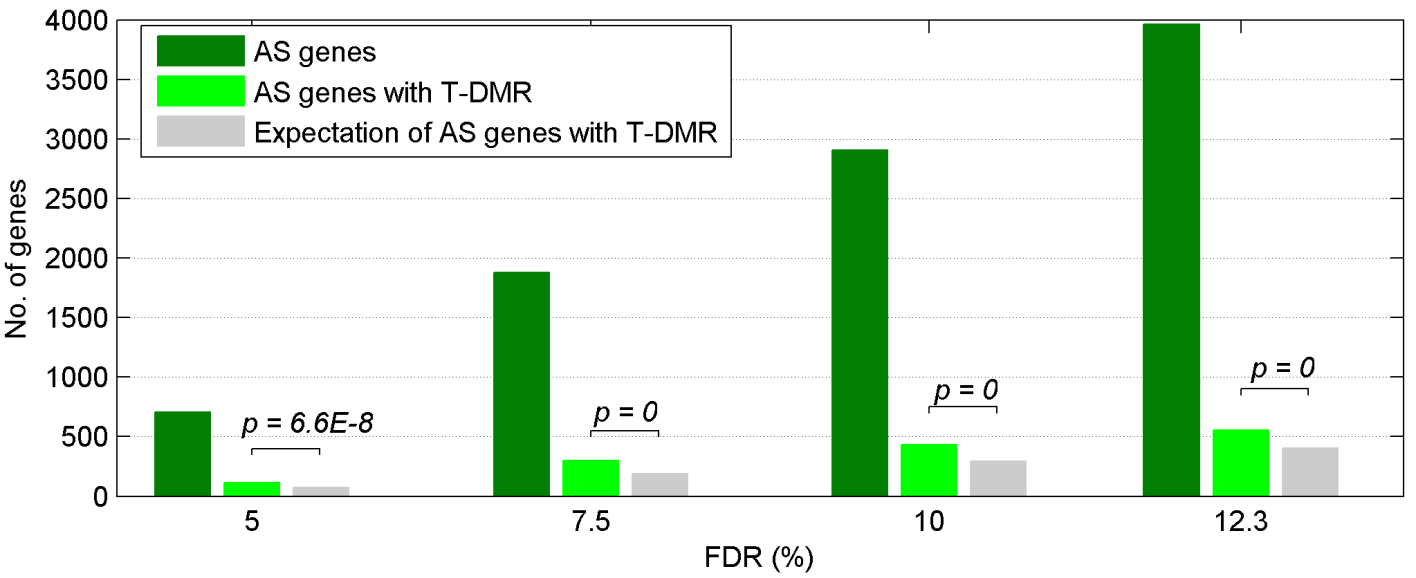


**Supplementary Figure 3.** Numbers of AS genes, AS genes with T-DMRs, and expected numbers of AS genes with T-DMRs at several different FDR cut-offs for alternative splicing. The enrichment of AS genes with T-DMRs was robust regardless of the cut-off selected.


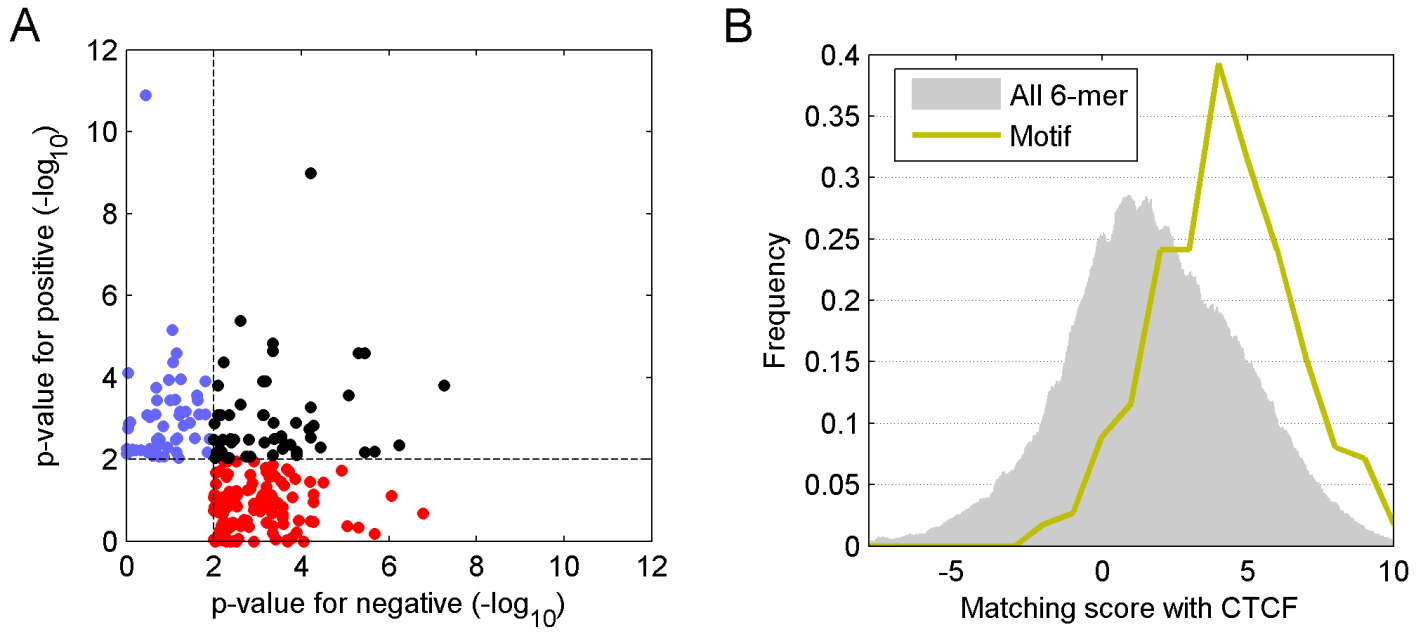


**Supplementary Figure 4.** The motif discovery in the T-DMRs upstream relative to the alternatively spliced exon. A) Significant motifs. Each dot represents a significant 6-mer motif. Motifs with different methods of regulation (red: negative; blue: positive; black: both negative and positive). X- and Y-axis are the values of –log(p) in negative and positive group, respectively. B) Similarity between predicted motifs and the CTCF consensus sequence.

**Supplementary Table 1.** Pyrosequencing primers for rat and mouse *Gm5607* conserved T-DMR.

| **Species** | **Forward Primer** | **Reverse Primer**  **(5ʹBiotinylated)** | **Sequencing Primer** |
| --- | --- | --- | --- |
| Mouse | AAGTGGAAGGTATAGTTTTTGATTAA | CCAAATTACTATTTCTATTCCTTCCTAATC | ATATGGGTAATTAGTATTAGTTTAT |
|  |  |  | GTTATAGGAGATTATGAAGATAGA |
|  | ATGTGTTAAATTAGATTAGGAAGGAATAGA | ACAACCCCACTCATTCCAA | AGTAATTTGGTTAATAAATTTTT |
|  |  |  | AAAATTTTATTTATTTTTGAA |
| Rat | GTTATTGGTTTTGGTTTGTTATGTGTA | ATTCCTTCCTAATCTCTTTTAACATATTC | ATATGGGTAATTAGTATTAGTTTAT |
|  |  |  | AGGAGATTATGAAGATAGAT |
|  | ATATGTTAAAAGAGATTAGGAAGGAATAGA | CACTCATTCCAACCACTCTC | AAATAGTAATTTGGTTAATAAATTT |
|  |  |  | AGGAATAAAATTTTATTAATTTTTG |

**Supplementary Table 2.** The number of positive and negative regulation events observed and their relationship with differential methylation in the retina and brain. Positive regulation (increased methylation + increased **inclusion**, e.g. ΔM > 0 & SI > 0, or ΔM < 0 & SI < 0) are highlighted in gray. Examples of negative regulation (increased methylation + increased **exclusion**) are not highlighted.

| Splicing Index (SI) Direction | ΔM > 0  *(hypermethylation in brain)* | ΔM < 0  *(hypermethylation in retina)* |
| --- | --- | --- |
| SI > 0  *(more inclusive in brain)* | 357 (24%) | 323 (21%) |
| SI < 0  (*more inclusive in retina*) | 469 (31%) | 366 (24%) |
